# Medical Prognosis of Infectious Diseases in Nursing Homes by Applying Machine Learning on Clinical Data Collected in Cloud Microservices

**DOI:** 10.3390/ijerph182413278

**Published:** 2021-12-16

**Authors:** Alberto Garcés-Jiménez, Huriviades Calderón-Gómez, José M. Gómez-Pulido, Juan A. Gómez-Pulido, Miguel Vargas-Lombardo, José L. Castillo-Sequera, Miguel Pablo Aguirre, José Sanz-Moreno, María-Luz Polo-Luque, Diego Rodríguez-Puyol

**Affiliations:** 1 Foundation for Biomedical Research, Hospital Príncipe de Asturias, 28805 Alcalá de Henares, Spain; alberto.garces@ufv.es (A.G.-J.); j.sanz@uah.es (J.S.-M.); 2 Center for Studies and Innovation in Knowledge Management, Universidad Francisco de Vitoria, 28223 Madrid, Spain; 3 Department of Computer Science, Universidad de Alcalá, 28805 Alcalá de Henares, Spain; huriviades.calderon@utp.ac.pa (H.C.-G.); jose.gomez@uah.es (J.M.G.-P.); jluis.castillo@uah.es (J.L.C.-S.); 4 E-Health and Supercomputing Research Group, Technological University of Panama, Panama City 0819-07289, Panama; 5 Ramón y Cajal Institute for Health Research, 28034 Madrid, Spain; mariluz.polo@uah.es; 6 Department of Technologies of Computers and Communications, Universidad de Extremadura, 10003 Cáceres, Spain; jangomez@unex.es; 7 Department of Electrical and Electronic Engineering, Technological Institute of Buenos Aires, Buenos Aires C1437FBG, Argentina; maguir@itba.edu.ar; 8 Department of Nursing and Physiotherapy, Universidad de Alcalá, 28805 Alcalá de Henares, Spain; 9 Department of Medicine and Medical Specialties, Research Foundation of the University Hospital Príncipe de Asturias, IRYCIS, Universidad de Alcalá, 28805 Alcalá de Henares, Spain; diego.rodriguez@uah.es

**Keywords:** early diagnosis, infections, patients, machine learning, computer systems, internet use, cloud computing

## Abstract

Background: treating infectious diseases in elderly individuals is difficult; patient referral to emergency services often occurs, since the elderly tend to arrive at consultations with advanced, serious symptoms. Aim: it was hypothesized that anticipating an infectious disease diagnosis by a few days could significantly improve a patient’s well-being and reduce the burden on emergency health system services. Methods: vital signs from residents were taken daily and transferred to a database in the cloud. Classifiers were used to recognize patterns in the spatial domain process of the collected data. Doctors reported their diagnoses when any disease presented. A flexible microservice architecture provided access and functionality to the system. Results: combining two different domains, health and technology, is not easy, but the results are encouraging. The classifiers reported good results; the system has been well accepted by medical personnel and is proving to be cost-effective and a good solution to service disadvantaged areas. In this context, this research found the importance of certain clinical variables in the identification of infectious diseases. Conclusions: this work explores how to apply mobile communications, cloud services, and machine learning technology, in order to provide efficient tools for medical staff in nursing homes. The scalable architecture can be extended to big data applications that may extract valuable knowledge patterns for medical research.

## 1. Background and Objectives

The world’s older population is growing at a significant rate. Today, 8.5% of the population is aged 65 and over; this will increase to 17% by 2050 [1]. Infectious diseases are common (and serious) in this group, often requiring the use of emergency services, degrading their efficiency, and sometimes overburdening them to the point of collapse [2].

The use of digital technology for health supports a patient-centric approach, based on communication, empathy, and collaboration between patients and practitioners [3]. eHealth provides mobility with portable devices, wearables or smartphones, ubiquitous services, and adaptive resources with the cloud. eHealth research addresses electronic records, computerized order entries, e-prescribing, clinical decision support systems (CDSS), telemedicine, health knowledge management, triage, virtual healthcare teams, and medical mobility services (mHealth). eHealth is supported by advanced IT, such as big data, machine learning, artificial intelligence, cloud services, mobile devices, and the internet of things (IoT) [4,5,6].

However, technology implementations could clash with practitioners and nurses in some cases, as they entail additional workloads. In addition, eHealth decision support systems do not absolve doctors from their accountability [7]. Data-based systems also face the challenge of scarce and poor-quality data in regard to training the model, sometimes becoming unaffordable due to ignoring procedures [8]. The long learning curve of a new eHealth system slows deployment; changes can also be seen as a threat to existing job conditions.

The hypothesis is that anticipating an infectious disease diagnosis by a few days could improve a patient’s well-being, alleviate the health system’s resources, and result in relatives having a “better perception”. Cost-effectiveness is an important driver for national health systems, i.e., to improve medical services for citizens [9]. The evolution of an infectious process is characterized by changes in vital signs. These data could be used to determine the probability of developing an infectious disease [10].

Machine learning (ML) techniques can be used for predictive modeling [11]. They provide pattern recognition and forecast the evolution of a disease, improving the protocols of control and care. ML is an area of computer science derived from artificial intelligence. It provides satisfactory results in eHealth and medicine [12].

This research presents the application of mobile communications, cloud services, and machine learning technology to provide efficient tools to medical staff in nursing homes in order to predict the development of infectious diseases. The approach taken by our study makes it somewhat different from other proposals, due to its particular characteristics. The patients were not cared for in usual health centers, but were elderly people living in nursing homes. Three particular infectious diseases were considered: acute respiratory, urinary tract, and skin and soft tissue infections; a customized biosensor system was developed for the project; the communications infrastructure for data collection, storage, and analysis ws based on microservices; and machine learning algorithms were integrated into the microservices for prediction purposes.

To this end, vital signs from the residents were taken daily and transferred to a database in the cloud by means of an experimental data capture system. Classifiers were used to recognize patterns in the spatial domain process of the collected data. In this context, this research found the importance of certain clinical variables in the identification of infectious diseases, as we will discuss later. These vital signs were selected because they may change due to the pathophysiological adaptations that take place in infectious diseases. Thus, the infection-related inflammation process, stress induced activation of the sympathetic nervous system, and modifications of the activities of the nuclei that regulate heart and lung functioning may modify body temperature, electrodermal activity, oxygen saturation, heart beat rate, and blood pressure.

## 2. Related Work

The references listed below were selected in relation to the underlying problem of this study: the possibility of early diagnosis by collecting and processing medical data that were further analyzed by advanced hardware and software architectures and tools.

### 2.1. Early Diagnosis

Given the importance of anticipating the diagnoses of infectious diseases, it is surprising that this concept remains a novelty. Similar approaches for high-risk and very severe cardiopathies [13] or pneumopathies [14] have been proposed. However, anticipating respiratory and urinary infections has not yet been practicable or sustainable, even after considering the high prevalence. The selected infectious diseases based on their prevalence in elderly individuals, are acute respiratory infection (ARI), urinary tract infection (UTI), and skin and soft tissue infection (SSTI).

### 2.2. Collecting and Processing Medical Data

Sensors used to capture medical signs can be placed in public or private spaces, such as cameras, barometers, microphones, passive infrared (PIR), ultrasound motion detectors, or radio frequency identification (RFID). They can also be embedded in mobile devices, such as accelerometers, magnetometers, or gyroscopes (A/M/G), and be worn, such as smartwatches or alert necklaces [15]. The last category of sensors are those expressly applied on the body of the patient, such as body thermometers, pulse oximeters, tensiometers, electrocardiograms (ECGs), or electroencephalograms (EEGs). The sensors can be networked through wireless sensor networks (WSNs) or body area sensor networks (BASNs).

Once heterogeneous clinical data are collected, they are cleaned, filtered, individualized, and combined. The use of ML, deep learning (DL), artificial intelligence (AI), or ambient intelligence (AmI) in this context [16] motivates research on the automatic identification of the basic activities of daily living. Service provisioning requires self-contention to ensure nonintrusive technology [17].

The information gathered by large sensor networks, such as the IoT [18], makes the utilization of multi-agent management integration [19] and pervasive mobile communications [20], known as mHealth in the medical field, advisable. This allows for delivering new advanced services, such as ECG wearable devices [21], accepted portable mobile applications [22], mobile advisors for drug dosage and adverse reactions [23], medical recommenders for different medical specialties [24,25,26], fast automatic triage [27], professional medical education programs [28], and telecare systems [29], among many others.

### 2.3. Hardware and Software Architectures and Tools

The cloud supplies computing power and data storage on demand as scalable commodities under three layers of service: infrastructure (IaaS), platform (PaaS), and software (SaaS). Security and privacy are key requirements for the cloud due to the sensitive information in medical records [5,30].

Microservice architecture is derived from service-oriented architecture (SOA) [31] to provide flexible and scalable execution properties in the cloud. Each microservice plays specific roles depending on the database (DB) requirements [32]. External configuration, microservice discovery, load balancing, central login, metrics, or autoscaling require attention. There are powerful software tools available for delivering applications rapidly by adopting the microservice paradigm [33].

With regard to software tools, ML provides a good approach for data-based predictive analysis [11]. Many algorithms provide knowledge pattern recognition and forecasting, suggesting the possibility of anticipating the diagnosis of diseases from monitored medical data. ML has techniques that have been widely applied, with satisfactory results in eHealth [12].

Some unsupervised learning techniques used in eHealth are K-means, density-based spatial clustering of applications with noise (DBSCAN), self-organized maps (SOMS), similarity network fusion (SNF), perturbation clustering for data integration and disease subtyping (PINS), and cancer integration via multikernel learning (CIMLR), among others. Common supervised learning algorithms in this domain are support vector machine (SVM), iterative dichotomizer 3 (ID3), K-nearest neighbor (KNN), Naive Bayes (NB), Bayesian networks, linear regression, and logistic regression for classification [34].

## 3. Materials and Methods

This research provides a complete experimental system for gathering medical information from elderly individuals, analyzing the data with predictive ML models.

The experiments performed follow a holistic view, where the data gathering requires defining the scope of the experiment, the protocol or workflow for the medical personnel, the mobile set of instruments, the software platform, and the data analysis techniques.

### 3.1. Participants, Procedure, and Ethical Considerations

This proposal is intended to be applied for institutional residents in nursing homes that are susceptible to developing infectious diseases. A workflow is defined for nurses to collect vital signs on a daily basis from their assigned residents. Doctors must report any infectious disease detected in any of these residents.

The protocol requires that residents or relatives approve its use beforehand due to normative restrictions and to protect the individual’s privacy.

**Figure 1:**
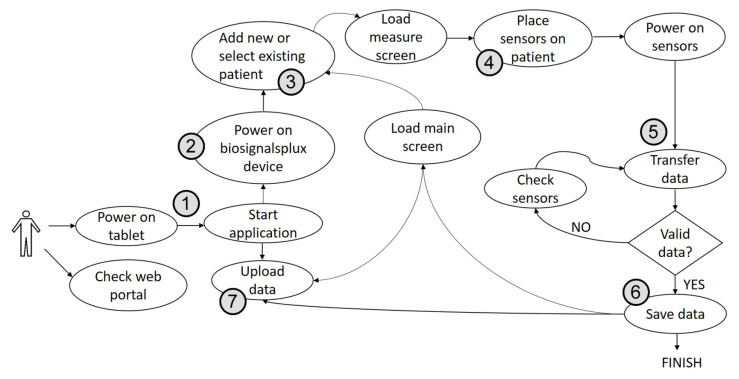
Protocol workflow.

The assigned nurses must follow the following protocol: seated in front of the resident, they (1) turn on the app (on the tablet); (2) switch the hub on, as well as the app connection; (3) select the patient’s ID; (4) deploy the medical sensors on the arms and hands; (5) press the start button to start the readings; (6) save the data locally once the measurements are verified; (7) and upload the data to the Cloud DB as soon as the tablet is connected to the internet (WiFi or telephony). Finally, nurses place the devices back into the cases for the next residents. Figure 1 shows the workflow of the procedure.

The nurses are to ensure that the batteries of the active sensors, hub, and tablet, are fully charged for the next day, and to promptly report any incidents observed that could compromise the data acquisition.

Finally, ethical consideration for setting clear limits for the research and protecting people’s privacy was implemented at the beginning of this project, following the instructions of the founders by means of private statements in their hands.

### 3.2. Instruments

The nurses use a portable set of biosensors to take the required medical signs from the residents. The equipment must be comfortable to carry and fast to deploy. The different components must be resistant to manipulation, and the application must be robust to perturbances and anomalous events. For general deployment, the equipment must receive approval from the respective health systems.

It is necessary that the equipment be seen as comfortable to operate by the assigned personnel and allow for a warm relationship with the resident. The mobile application that operates the equipment must be fully functional online and offline, avoiding delays in the process.

**Figure 2:**
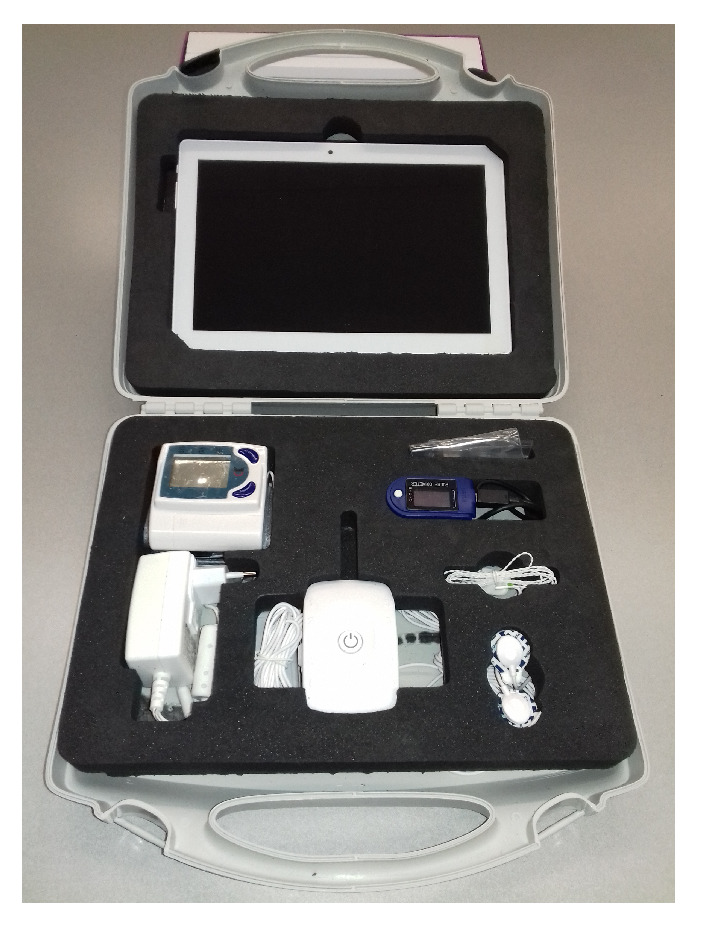
Customized briefcase to carry the medical sensor set.

The field equipment used by the nurses are small cases with customized sets of four sensors obtained from a commercial vendor [35], prepared for the devices and the tablets used for the mobile applications, as shown in Figure 2.

**Figure 3:**
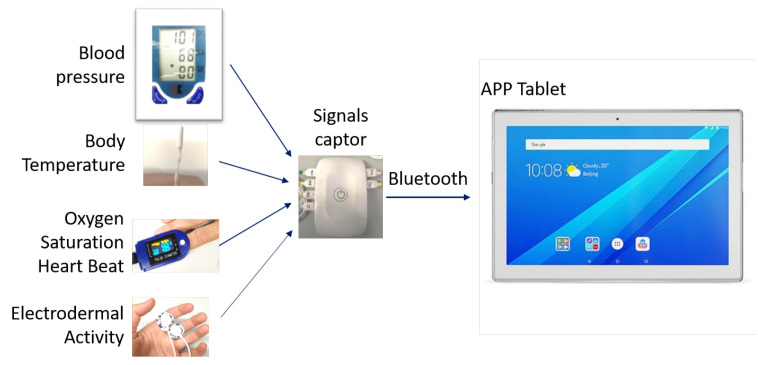
Medical sensor, hub, and android tablet connections.

The four biosensors collected five vital signs, taking (1) the average, maximum, and minimum values of the electrodermal activity (EDA) and heart beat rate; (2) the maximum and minimum oxygen saturation (SPO2); (3) the body temperature when stable; and (4) both the systolic and diastolic blood pressure. A hub quantifies and multiplexes the signals and sends them as a mobile application (app) via Bluetooth, as shown in Figure 3.

After preprocessing the signals, the android-based apps check that the values are within the expected ranges, store them in the tablet, and try to connect to the internet when WiFi is available to upload the outstanding records asynchronously to the cloud DB. The operation does not stop when there is no internet coverage. If there are 19 variables (means, dates, flags, etc.), and the device needs to reserve memory, considering the same size for all residents (double precision), and each device is used with 100 residents monitored daily, the local memory required over 2 years would be 84 MB. The current version of the app requires 50 M, which is far less the resources of the tablet, i.e., 11 GB [36], or any current mobile phone.

The hub autonomy is 10 h in streaming mode, and the tablet has a 5000 mAh battery that lasts approximately 3 h, which is enough for one day if it is fully charged overnight. Cables are a problem, however, and the main cause of failure, as they are too thin, and the nurses are in a hurry. Wireless connections for the sensors should work better.

### 3.3. System Design

This project requires special attention to the software platform, implemented by cloud services and microservice architecture. The software platform must provide flexibility in computation resources and functionality to quickly adopt new services and applications. Collaboration among different teams must be ubiquitous, and the nature of the data leads to a need for special attention to its security. The cloud suits the requirements well, but it is necessary to respond to security, availability, maintainability, and normative issues [37].

Two main scenarios must inspire the deployment of the software platform: (1) for offline applications, such as medical decision assistants, and (2) for online applications, such as remote real-time monitoring telecare, which encompass the need for immediate actions triggered by the alerts [38].

The data stored in the mobile application are exported in CSV format to a database in the cloud and then remain available for the micro-service-based data analysis tasks.

The SaaS database is asynchronously nurtured from quasi-unlimited concurrent uploading sessions set with each mobile application. A small cloud storage package of 100 GB would support 1190 mobile applications, monitoring 100 residents each, uploading data for 2 years, yielding a total of 119,000 residents. The cloud provides [39] (1) efficient multitenancy; (2) sharing data for different goals; (3) elastic scalability, allowing rapid deployments of complete scenarios, in nonstop ongoing synchronization and self-adapting to the new demands of resources; and (4) data privacy.

The cloud software [40] is implemented in a layered microservice architecture [41], because of [42] (1) the low-cost implementation; (2) the options available to develop different levels of software quality; (3) the scalable and adaptable resource configuration on demand—each component can be individually duplicated; and (4) the compatibility with smart devices and several communication protocols. Mobile devices access the API gateway and user PCs with the web UI. Users call the microservices with their credentials (“nurse”, “doctor”, or “administrator”). The microservices interconnect themselves to build a single application for users.

**Figure 4:**
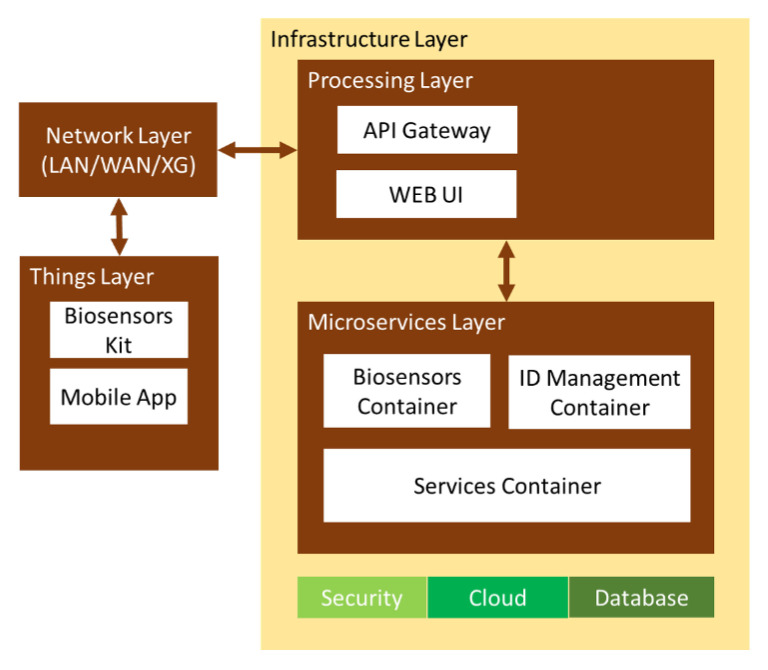
Microservices software architecture for anticipated diagnosis of infectious diseases.

The microservices are classified into three groups, all sharing the data via JSON request/response HTTP for (1) interfacing with the physical biosensor application; (2) managing the access policies; and (3) synchronously recording the reports by doctors about patients developing infectious diseases. The architecture uses the representational state transfer (REST) API for data integration, transference, and storage [43]. The software applications are split into small purpose-specific programs with UIs for different domains or APIs to interconnect with third-party applications [44] throughout the infrastructure layer [32], as shown in Figure 4.

The “things” layer connects the hardware to the network layer and validates the signals. The network layer opens connections to mobile applications for asynchronous data transference [45]. The processing layer assigns each device the corresponding access privileges for their methods to the microservices. The microservices layer provides the service respontses to the specific queries. The infrastructure layer delivers availability, scalability, and data integrity for the upper layers, with networking, processing, and storage resources [46]. It manages data with MapReduce in distributed computing [44] and stores data with Apache Hadoop in Cassandra NoSQL DB [47] to improve the error tolerance. This layer also accepts complex computation tasks from upper layers—authentication or interoperability—to alleviate their resources for their primary operations [48].

### 3.4. Analysis by Machine Learning Classifiers

The daily medical data gathered from residents can be treated with ML techniques in time or spatial domains, also known as longitudinal or cross-sectional studies. The time approach studies the evolution of certain variables using time series, while the spatial approach considers the relations among the medical variables of a sample, finding patterns and making classifications, according to all samples. Time series are good for predicting, but are not essential. In fact, the spatial domain is more precise for pattern recognition analysis. The spatial domain works complementarily with the time domain approach.

In this work, we approached the analysis of the clinical data under a spatial approach. The absence of the time component prevents a predictive approach, but the spatial dimension allows for a more precise pattern recognition analysis. In this way, it is possible to classify a sample measured from an individual as a sample recognized as possibly indicating an infectious disease. In addition, the information obtained with this approach will allow us to perform a better predictive analysis based on time series in the future.

The learning phase allows us to know the relative weight of each direct or transformed feature in the classification or prediction of an infectious disease. The performance of the classifier can be measured with error performance and the coefficient of determination ($R^{2}$).

This analysis is coded with an API of the Waikato Environment for Knowledge Analysis (WEKA) [49], Java-based ML software implemented in the Apache Spark development environment [50], Java Servlet (JS), and Java Server Pages (JSP) [51] for handling the data entries of the microservices. The Java web service is in Apache Maven [52].

The goal of applying these ML methods was not to compare the prediction rate with the true incidence, but to be a first approach for data classification integrated on the web service, to experiment with the influence of the clinical variables on the predictable yielding success with regard to each type of infection.

## 4. Results

Spanish health authorities approved running this project in two nursing homes in Madrid. The main population and resources of these institutions are shown in Table 1. The data in the table refer to residents participating, where the inclusion criterion was the ability to understand the purpose of the experiment and volunteering.

The medical team selected the variables listed in Table 2 and indicated their expected ranges.

The mobile application attaches the date and time of the sample and the patient identification code to the collection of signals; this is manually anonymized by the nurse assigning the code, and the nurse sets flags to indicate if the record has been successfully uploaded.

### 4.1. Protocol and Acceptance

The healthcare personnel (doctors and nurses) are trained to know the process, look after the equipment, and fix minor incidents. The learning process was conducted with 18 volunteers until they could proceed autonomously. After that, they were requested to simulate, more than once, the process for taking samples and recording the duration. The purpose of this small experiment was to improve the data collection protocol, not only to facilitate the work of the personnel, but also to increase the potential number of residents susceptible to monitoring. Table 3 shows the results.

Learning the process takes only 7 min on average. The vital sign collection, on the other hand, only takes 4 min and 15 s on average, yielding the possibility for one nurse to monitor 54 patients in 4 h. Greetings and moving to the next room, along with any other activity could slow down that rate, although practice would compensate and speed up the process.

The experiment also recorded the volunteers’ ages, ranging from 20 to 70 years, and digital competency with a self-graded scale from 1 (IT illiterate) to 5 (digital native). Older volunteers spent slightly more time learning than younger volunteers, but the Kruskal–Wallis (KWT) [53] test gave a *p*-value of 0.428, showing that this result is not conclusive. The KWT—applied for the need to be IT skilled—gave a *p*-value of 0.088, confirming that the personnel do not need to be IT literate.

The process is fast and comfortable, as only the arms and hands of the residents are exposed to the daily test, not requiring undressing or intimate contact. Additionally, the presence of the nurse helps to generate a friendly environment for caregivers and residents.

The protocol for the doctors does not require anything other than reporting when the resident is developing a disease, which infection the symptoms are compatible with, the date of the alert, and if there was a referral to the emergency services.

### 4.2. System Efficiency

The software architecture attempts to check its efficiency, integration, compatibility, and performance, counting errors when the components interact [54]. The testbed has two Docker containers (1 vCPU, 4 GB, 120 GB Disk, Ubuntu 16.04.6 LTS); Apache for the endpoint and Nginx as the frontend proxy and load balancer. Nginx receives the user queries and forwards them to the microservices; Apache JMeter measures the workload [55]. BlazeMeter servers (US East—Virginia, AWS) simulate the workload [54,56]. Microservice #1 (MS1) provides a search of over 6297 records, and Microservice #2 (MS2) requests 138 records of residents with infectious diseases for 20 and 50 concurrent virtual users. ALL is the sum of MS1 and MS2. There are two scenarios: EIM-1-FB with frontend–backend “full stack”, shown in Table 4, and EIM-2-F, frontend-only, shown in Table 5 and two workload tests of 20 min each with 20 and 50 virtual users (threads), respectively, which gradually increase the queries per second, reaching 14,602 on average.

### 4.3. Data Analysis

This research analyzed the performance of three space-based ML algorithms: (1) naive Bayes (NB) [56], which is easy to implement and is widely used for medical diagnosis and disease prediction; (2) filtered classifier (FC) [57], which classifies previously filtered or preprocessed data; and (3) random forest (RF) [58], a supervised classifier that is used for this work the random tree (RT) technique to build decision trees for the classification. The reason for choosing these three algorithms is that they allow modifications of the weight of the attributes. In this way, the medical personnel can assign different significance levels to the clinical parameters for diagnostic purposes; for example, we can assign more relevance to body temperature in the classification and analyze how the corresponding results improve (or not) in other cases. The basic settings of these algorithms were those provided by default in WEKA to handle problems of similar complexity.

**Figure 5:**
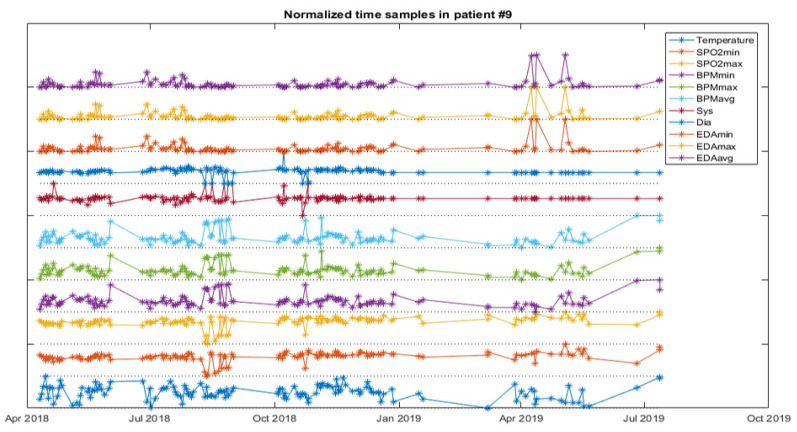
Vital signs evolution of one resident with ARI.

**Figure 6:**
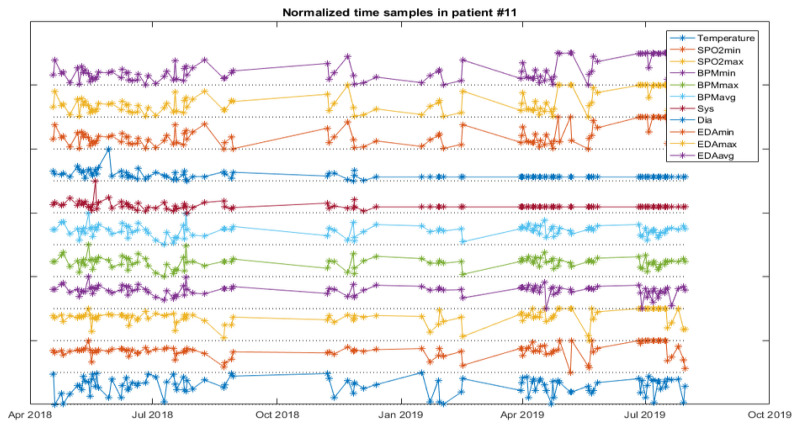
Vital signs evolution of one resident with UTI.

**Figure 7:**
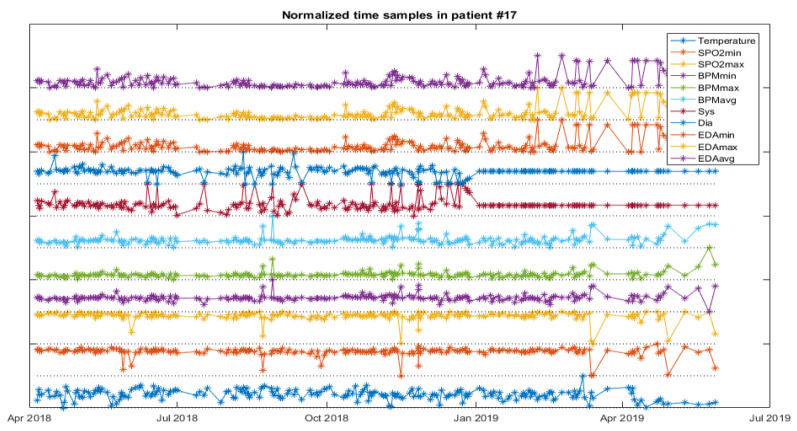
Vital signs evolution of one resident with SSTI.

The analysis starts with a web service for NB and FC as a first approach for data classification, and then a software application to experiment with the weights of the variables for the RF is developed. The next figures help to visualize the data of patients developing infections. Figure 5 depicts the vital signs of one resident developing IRA, Figure 6, one developing a UTI and Figure 7, another developing an SSTI.

The WEB service for NB and FC classifies an input sample as a possible ARI, UTI, or SSTI infection. First, the user selects the algorithm and then uploads the medical data record of any resident. The trained model classifies the test data into the three types of infection, providing, as a result, the success rate of the classification (% for the patient developing any of the infectious processes and identifying which one it is) and the weight of each medical parameter affecting the prediction. The model has been trained with other residents who have developed these diseases.

## 5. Discussion

The obtained results are quite satisfactory, but they are not accurate enough due to the short period for sampling. To obtain more accuracy, it is necessary to either extend the period of the experiment or apply certain techniques for small datasets, such as including context variables, such as the season, holidays, and weather [59].

**Figure 8:**
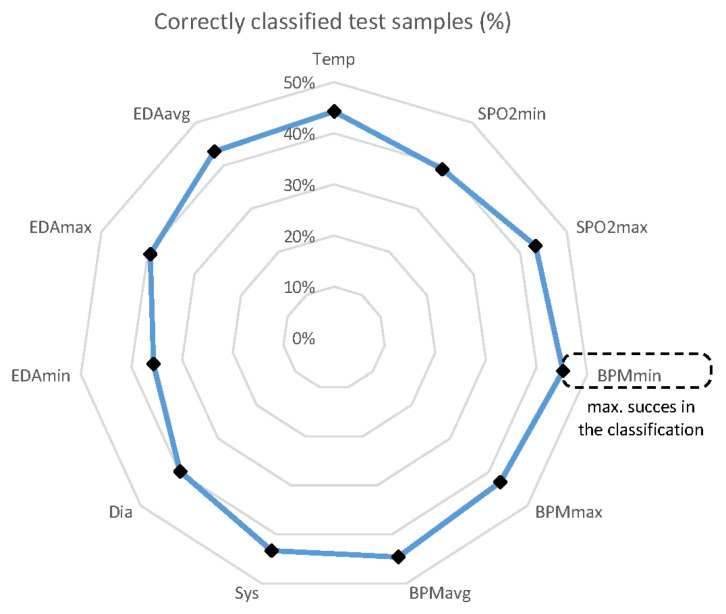
Relative frequency of correct classifications per variable.

With RF, the type of infectious disease is deduced from the independent variables, considering low entropy, a measure of the amount of possible information disorder or randomness. All available samples (6277) from all patients (60) are split 95% for the training dataset (5963) and 5% for the validation dataset (314). Only 129 developed ARI, 95 UTIs, and 90 SSTI infections. The process is repeated up to 11 times, changing the weights of the variables according to their importance in the classification. Thus, it is possible to determine which of the 11 variables is better for medical personnel to concentrate on when monitoring the patients. With the available data, the minimum heartbeat rate (HBR) provided the best success rate (%), followed by the average HBR, as shown in Figure 8.

**Figure 9:**
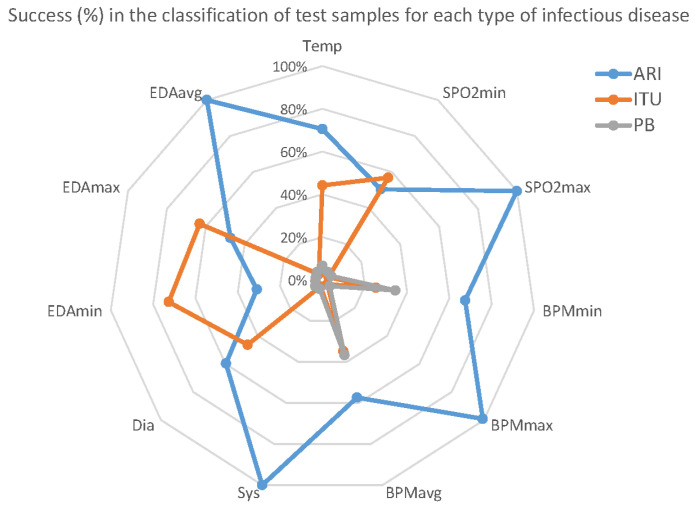
Success ratio detecting infectious diseases. (a) Orange: ITU, (b) Gary: PB, (c) Blue: ARI.

Although these figures do not show an impressive performance prediction in general, the results present a significant difference in predictability for each infection. Figure 9 shows the true positives (%) with respect to the variables for each of the diseases.

ARI infection is significantly more predictable, yielding 100% success for some variables. On the other hand, SSTI infections are harder to predict with the selected variables. It is necessary to note that there are fewer SSTI training records than for ARI, and the former model was less trained than the latter, again indicating the need to extend the period of sampling.

Finally, the most important variable for all of the diseases is the average HBR.

## 6. Conclusions

This research proposes a comfortable, flexible, accessible, and cost-effective eHealth monitoring system for residents in nursing homes, and analyzes the predictability of infectious diseases based on the vital signs collected in the pilot study. Its cost-effective implementation allows disadvantaged areas and less accessible populations to be reached.

This paper has demonstrated that the system is easy to use and that there is no need for IT skills for nurses. In addition, the protocol is especially resident-friendly, improving relationships with caregivers.

The microservice-implemented architecture is cost-effective and scalable. The stress tests indicate when the system experiences saturation. The functionality of the microservices to carry out the optimal service has been tested. The mobile application is an open standard that can be installed in any android device with minimum changes.

The problems of the cable organization are under research, with a new design of wireless biosensors ported in a suitcase or a tray. The active elements will be charged on a contactless surface, releasing the nurses or replacing the exhausted batteries. The equipment will be approved by the authorities.

Regarding the data analysis, the main contribution of this work lies in the findings of the importance of certain clinical variables in the identification of infectious diseases. There was no variable with significant relevance for predicting all of the selected infectious diseases. However, among these diseases, the HBR showed a higher impact in the classification. Moreover, the ARI was proven to be better detected than the others. The size of the scenario conditions the classification results. Other factors that affect the predictions are the repeatability of the biosensors, the awareness of the medical personnel, and the type of infection (among other factors). However, this study shows some valuable results and is a good starting point for further research.

Other space- and time-based machine learning techniques will follow this study. In particular, it is interesting to tackle the clinical data analysis under a time series approach. Moreover, larger datasets should be collected to improve the classifier training and the time series accuracy.

## Figures and Tables

**Table 1:** Monitored population and resources.

Population	Cardenal Cisneros	Francisco de Vitoria	Total
Residents	127	316	443
Participants	20	40	60
Participants (%)	16%	13%	14%
Participants who developed disease	7	33	40
Minimum age	79	67	67
Maximum age	94	101	101
Average age	88.7	89.7	89.5
Std. deviation	5.1	7.0	6.6
Medical staff	4	14	18
Start collecting	24 March 2018	4 April 2018	
End collecting	11 March 2019	11 March 2019	

**Table 2:** Vital signs to monitor and life-compatible ranges.

Vital Sign	Valid Range	Out of Range
Body temperature (T)	34 °C < T < 42 °C	T < 34 °C, T > 42 °C
Electrodermal activity (EDA)	EDA > 0.2 µS	EDA < 0.2 µS
Oxygen saturation (SPO2)	70% < SPO2 < 100%	SPO2 < 70%
Heart Rate (HBR)	HBR > 30 bpm	HBR < 30 bpm
Blood pressure (DIA)	DIA > 30 mmHg	DIA < 30 mmHg
Blood pressure (SYS)	SYS > 60 mmHg	SYS < 60 mmHg

**Table 3:** Time (h:min:s) required for training and taking one sample.

Activity	Mean	Std. Deviation
Learning process	0:07:00	0:02:10
Process execution:		
Sensors deployment on the body	0:01:55	0:00:49
APP initialization	0:00:33	0:00:38
Sensors delay	0:01:12	0:00:33
Upload the data to the cloud and resume	0:00:35	0:00:22
Total time consumed per resident	0:04:15	0:01:14

**Table 4:** Workload testing report for EIM-1-FB 20/50. The time measurement is the average in ms.

Label	Samples	Resp. Time	Avg. Hit/s	90% Line	99% Line	#Error	Avg. Latency	Users
ALL	29,939	782	25	755	5247	0	252	20
MS1	14,977	811	13	767	5151	0	280	20
MS2	14,962	752	13	719	5311	0	223	20
ALL	29,227	2003	24	1863	13,759	0	582	50
MS1	14,625	1991	12	1863	13,439	0	627	50
MS2	14,602	2016	12	1871	14,079	0	538	50

**Table 5:** Workload testing report for EIM-2-FB 20/50. The time measurement is the average in ms.

Label	Samples	Resp. Time	Avg. Hit/s	90% Line	99% Line	#Error	Avg. Latency	Users
ALL	3456	6785	3	15,487	38,911	6	3520	20
MS1	1733	6777	1	15,295	37,375	5	3645	20
MS2	1723	6794	1	15,487	40,703	1	3582	20
ALL	215,825	164	180	53	1047	79,944	85	50
MS1	107,925	147	90	56	1047	39,973	90	50
MS2	107,900	182	90	49	1047	39,971	79	50

## Abbreviations

The following abbreviations are used in this manuscript:

AI	Artificial Intelligence
AmI	Ambient Intelligence
ARI	Acute Respiratory Infection
BADL	Basic Activities of Daily Living
BASN	Body Area Sensor Networks
CDSS	Clinical Decision Support Systems
CIMLR	Cancer Integration via Multi-kernel Learning
DB	Database
DBSCAN	Density-Based Spatial Clustering of Applications with Noise
DIA	Diastolic Blood Pressure
DL	Deep Learning
ECG	Electrocardiogram
EDA	Electrodermal Activity
EEG	Electroencephalogram
FC	Filtered Classifier
HBR	Heart Beat Rate
IaaS	Infrastructure as a Service
IADL	Instrumental Activities of Daily Living
ID3	Iterative Dichotomizer 3
JS	Java Servlet
JSP	Java Server Pages
KNN	K-Nearest-Neighbor
KWT	Kruskal–Wallis
mHealth	Medical Mobility Services
ML	Machine Learning
MSE	Mean Squared Error
NB	Naive Bayes
NIST	National Institute of Standards and Technology
PaaS	Platform as a Service
PINS	Perturbation Clustering Approach for Data Integration and Disease Subtyping
PIR	Passive Infrared
REST	Representational State Transfer
RF	Random Forest
RFID	Radio Frequency Identification
SaaS	Software as a Service
SNF	Similarity Network Fusion
SOA	Service Oriented Architecture
SOMS	Self-Organized Maps
SPO2	Oxygen Saturation
SSTI	Skin and Soft Tissue Infection
SVM	Support Vector Machine
SYS	Systolic Blood Pressure
UTI	Urinary Tract Infection
WEKA	Waikato Environment for Knowledge Analysis
WSN	Wireless Sensor Networks
